# The peptide AC 2 isolated from *Bacillus*-treated *Trapa japonica* fruit extract rescues DHT (dihydrotestosterone)-treated human dermal papilla cells and mediates mTORC1 signaling for autophagy and apoptosis suppression

**DOI:** 10.1038/s41598-019-53347-3

**Published:** 2019-11-15

**Authors:** Gun He Nam, Kyung-Jo Jo, Ye-Seul Park, Hye Won Kawk, Je-Geun Yoo, Jin Dong Jang, Sang Moon Kang, Sang-Yong Kim, Young-Min Kim

**Affiliations:** 1 Department of Biological science and Biotechnology, College of Life science and Nano technology, Hannam University, 1646 Yuseong-daero, Yuseong-gu, Daejeon 34054 South Korea; 2 Doori Cosmetics Co.,Ltd., 11F Galaxy Tower, 175, Saimdang-ro, Seocho-gu, Seoul South Korea; 3 ANPEP INC. 13, Oksansandan 1-ro, Oksan-myeon, Heungdeok-gu, Cheongju-si, Chungcheongbuk-do Republic of Korea; 4 Department of Food Science & Bio Technology, Shinansan University, Daehakro Danwon-gu, Ansan-city, Gyenggi-do South Korea

**Keywords:** Biochemistry, Biological techniques, Biotechnology, Cell biology, Chemical biology, Developmental biology, Drug discovery

## Abstract

The *Trapa japonica* fruit is a natural plant growing in ponds with its roots in the mud. It has long been used as a home remedy for many diseases; however, a major problem with this kind of natural extract is the multicomponents-multitargets for diseases. Such problems make it difficult to identify the mechanism of action. Another problem is quality control and consistency. The aim of this research was to isolate a single bioactive compound (peptide) derived from the *Trapa japonica* fruit. The research was conducted with various experimental techniques, such as fermentation and liquid chromatography, to isolate a peptide. We isolated the AC 2 peptide from *Trapa japonica* fruit and found it to be promising on human dermal papilla cells. Dihydrotestosterone (DHT) stresses human dermal papilla cells and is a major cause of hair loss resulting from hormones and environmental factors. The purpose of this research was to develop an understanding of the mechanism by which the AC 2 peptide rescues dihydrotestosterone (DHT)-treated human dermal papilla cells. We explored the effects of the AC 2 peptide on the cell biological functions of human dermal papilla cells (HDPs). HDPs were treated with the AC 2 peptide and DHT. Then, a cytotoxicity assay, flow cytometry, Western blot, immunoprecipitation, and 3D cell culture for immunohistochemistry were conducted to investigate the mTORC1 pathway and suppression of autophagy and apoptosis. In addition, we also synthesized the AC2 peptide as an alternative to the expensive and difficult isolation and purification procedures and confirmed its potential in biomedical applications. We also validated the effects of the synthetic AC2 peptide as well as the isolated and purified AC2 peptide and established their similarity. Although extensive research has been carried out on natural extracts, few single studies have isolated and separated a bioactive peptide (single compound).

## Introduction

Human scalp and hair perform multiple functions, such as external shock absorption, UV protection, and heat insulation, but they also play an important role in appearance^1^. Because healthy hair is a prerequisite for an attractive appearance, the market for hair loss and hair-related products has been rapidly expanding, with an abundance of related information on the Internet^2,3^. Despite the increased attention on hair conditions, medical professionals are relatively less interested in hair and scalp disorders. Further studies are needed to investigate hair-related diseases for the development of appropriate treatments for hair loss and enhanced awareness of hair conditions^4^.

Hair is synthesized via processes in the ectoderm of the skin and musculoskeletal mesoderm. Hair passes through the phases of growth, development and death as it exchanges signals with dermal papilla cells in the mesoderm at the bottom of the follicle as well as other surrounding cells^5,6^. When hair follicles are stimulated by signals from dermal papilla cells, hair undergoes continual splitting. If dermal papilla cells are defective due to irritation or hormonal imbalance, hair cannot grow normally^7^. This condition is referred to as hair loss. In other words, the proliferation and activity of dermal papilla cells are directly associated with hair loss^8^. New treatment strategies that accelerate the proliferation of dermal papilla cells are needed.

Due to increasing incidences of hair loss in the human population, the market for hair loss products is rapidly expanding. Finasteride and minoxidil are the only two drugs approved by the United States Food and Drug Administration (FDA) for hair loss^9,10^. Testosterone generates dihydrotestosterone (DHT), which is the major cause of hair loss when it reacts with 5**α**-reductase of dermal papilla cells^11,12^. Finasteride inhibits the activity of 5**α**-reductase to delay hair loss and induces hair growth; however, it is accompanied by numerous side effects, such as a hypoactive sexual desire, erectile dysfunction, low ejaculate volume, and gynecomastia^13^. Minoxidil acts on blood vessels and follicles to increase blood flow, nourishes dermal papilla cells, and enhances hair growth; however, it is also associated with side effects such as hypertrichosis, dry scalp, and scalp irritation^14^. In addition, minoxidil induces the growth of fine soft hair but hardly any thick hair. The effects of drugs such as finasteride and minoxidil, which are used in conventional hair loss treatments, immediately stop when their use is discontinued and so long-term treatment for hair loss should minimize the side effects^15^. Therefore, apart from chemical treatments, new natural hair loss medicines are needed to minimize the side effects and induce the growth of dermal papilla cells. However, a major problem with this kind of natural extract is the multicomponents-multitargets for diseases. Such problems make it difficult to identify the mechanism. Another problem is quality control and consistency^16^.

To address these problems, investigators have recently attempted to isolate bioactive compounds derived from natural plants^17,18^. However, the separation and isolation processes of natural products from plants is extremely complex, and few single studies that undergo these processes have been performed. Bioactive single compounds consist mainly of peptides, which are fragments that are encrypted in the primary sequences of proteins and confer functions beyond nutritional benefits related to cellular growth factors^19^. Additionally, the use of peptides as potential medicinal ingredients in the present pharmaceutical market is increasing due to their extremely low toxicity and high bioactivity^20^. However, peptide-based medicines constitute a small proportion of the entire pharmaceutical market. A majority of the pharmaceutical market comprises low-molecular weight medicines synthesized in a laboratory. To diversify the chemical tools for the design of enhanced peptide treatments, advanced methods for peptide synthesis need to be developed.

*Trapa japonica* fruit is a natural plant that grows in ponds with its roots in the mud. It has long been used as a home remedy for many diseases. Our previous study reported that the extract of *Trapa japonica* fruit shows the most promising cell proliferation effects on human dermal papilla cells. This indicates a need to investigate the bioactive peptides derived from the *Trapa japonica* fruit^21^.

It is difficult to obtain peptides as the main ingredient in conventional biomedicines because they are directly extracted from natural products via expensive and complex fermentation and separation processes. Therefore, this study demonstrated the effects of a natural peptide via exploration and isolation, suggested synthetic peptide mechanisms, and investigated their medicinal and commercial potential as novel medicines.

## Results

### Isolation of the AC2 peptide from *Trapa japonica* fruit

**Figure 1:**
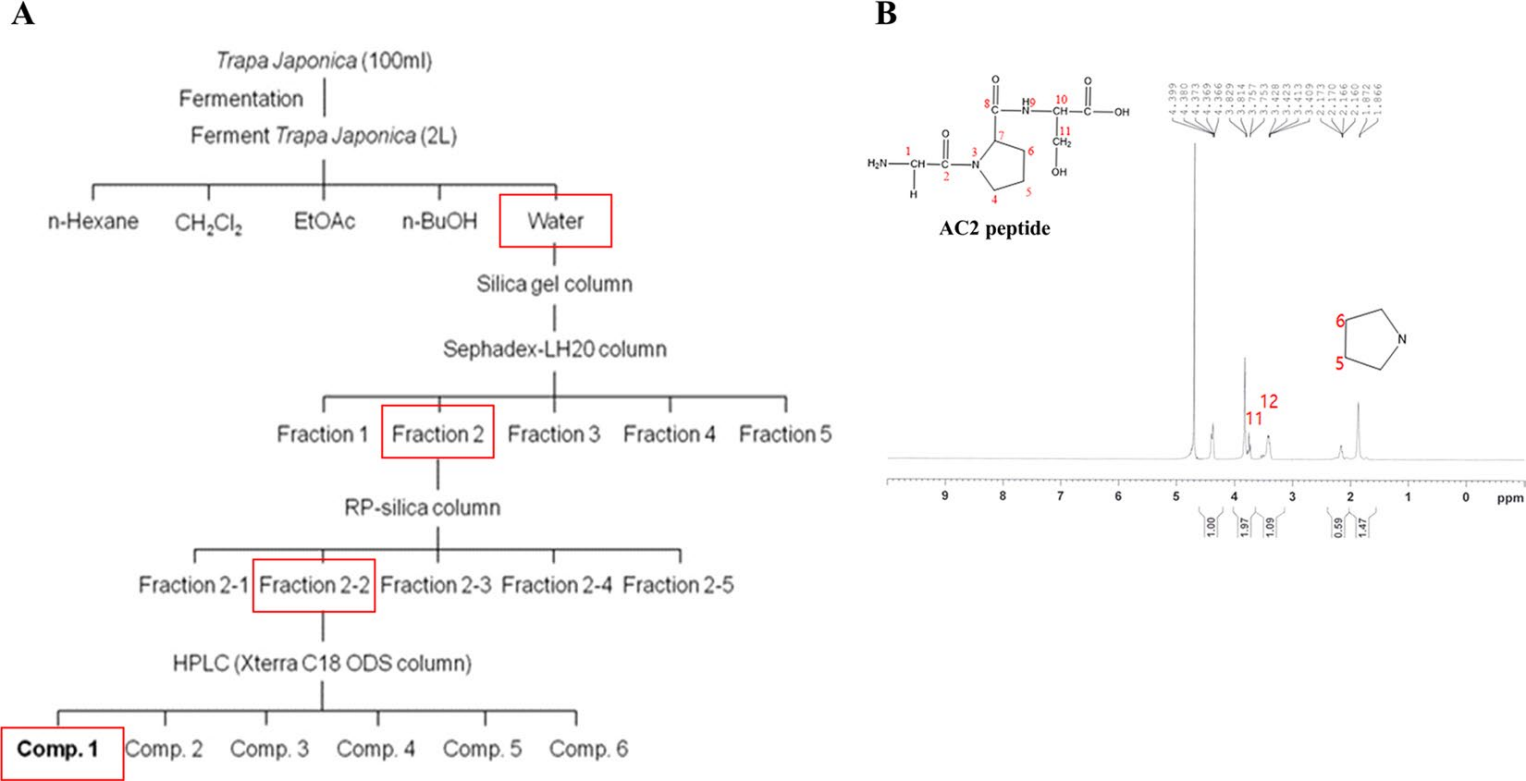
(**A**) preparation and *Isolation* of the peptide AC 2 from *Bacillus* treated *Trapa japonica Fruit* using various method (**B**) NMR profile of the peptide AC 2.

The fruit of *Trapa japonica* was separated and refined to separate the useful peptide from the ferment (Fig. 1A) to check for pure substances and to establish separation and refining conditions. To separate bioactive peptides, the peptide content in the fractions was checked (Table 1). When confirmed by fractionation after fermentation, the protein content increased 10-fold greater than the extract, and the conditions for separation and rectification were established in the following ways. The protein content measurement during the separation and refining process confirmed that the protein content increased by approximately 12.3% in the case of the dried ferment compared to the extract and the water layer had the most protein as a result of separating the ferment into hexane, CH2Cl2, EtOAc, N-BuOH and water layers. In addition, the water fraction was divided with separate resins using structural characteristics through the Sephadex-LH20 column and used for secondary purification of the separated fraction according to polarity. For further separation of the fraction separated by structural characteristics, a silica column was selected, and a RP-silica column suitable for separation of the water layer was used. Separation was performed through RP-silica in the second stage, and the fraction with the highest protein content was selected. After increasing the purity of the protein by HPLC, the peptide AC 2, which was a pure single peptide, was finally isolated among the six peaks. Finally, pure compounds were obtained through pre-HPLC. The structure of the pure compound was analyzed by NMR (^1^H, ^13^C, 2D-NMR) and confirmed as peptide AC 2 (Fig. 1B).

**Table 1:** Analysis of peptide and total protein content.

	Peptide content (mg/mL)	Total protein content (mg)
The hot water extract of *Trapa japonica* fruit	0.16	160.31
The fermentation of *Trapa japonica* fruit	0.18	180.15
Water layer	1.92	410.76
Sphadex-LH20 Fraction 2	2.27	410.23
RP-silica Fraction 2-2	2.56	514.99
HPLC Compound 2 (AC 2 peptide)	2.51	522.95

### Measurement of Cytotoxic activity and Cell cycle arrest

**Figure 2:**
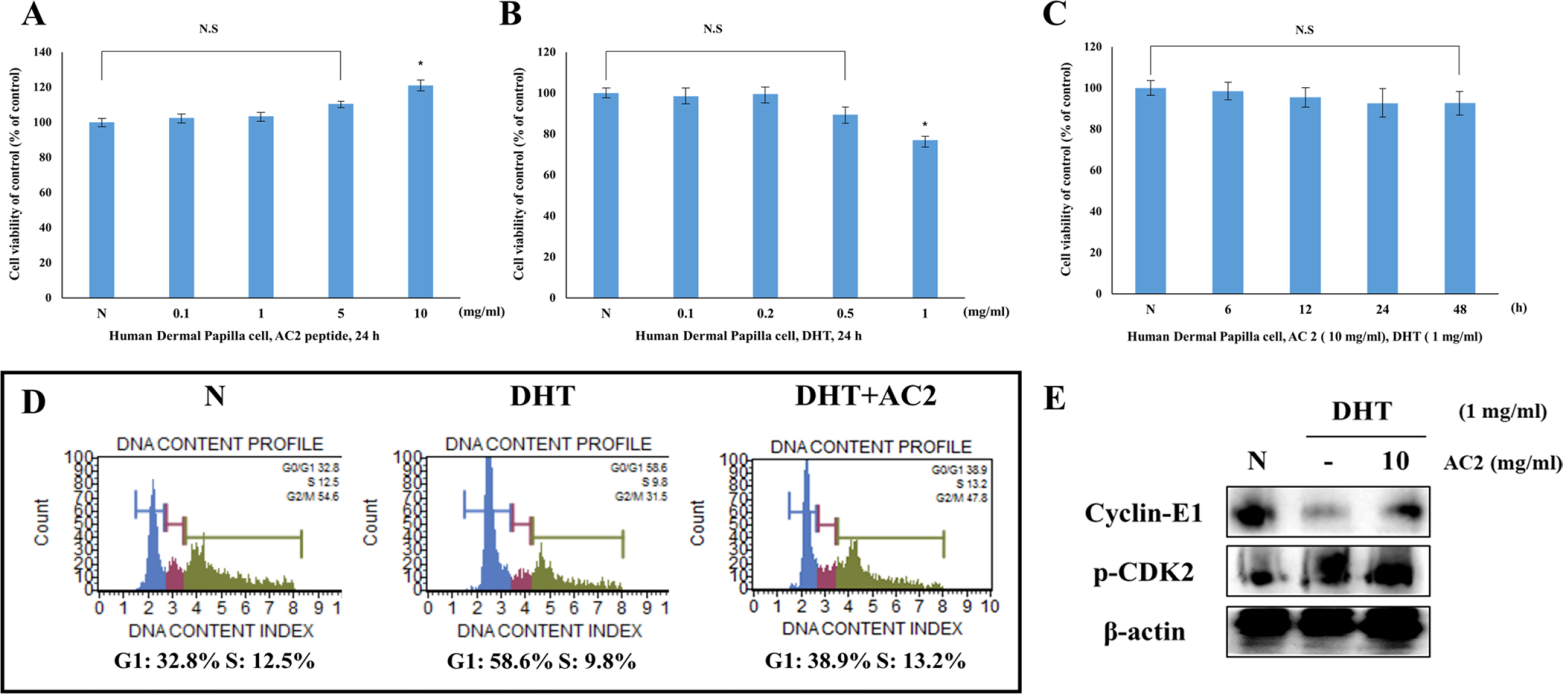
Measurement of Cytotoxic activity and Cell cycle arrest using WST-1 assay, flow cytometry and Western blot analysis (**A**) Cell viability was measured by WST-1 assay. Cells were treated to with variable concentrations of AC2 peptide (0.1–10 mg/ml) for 24 h. (**B**) Cell viability was measured by WST-1 assay. Cells were treated to with variable concentrations of DHT (0.1–1 mg/ml) for 24 h. (**C**) Cell viability was measured by WST-1 assay. Cells were co-treated to with AC2 peptide (10 mg/ml) and DHT (1 mg/ml) for various time (6–48 h). (**D**) AC2 peptide occurs cell cycle progression and inhibits the DHT-induced cell cycle arrest. Cell cycle arrest effect was measured by flow cytometry. (**E**) Cell cycle-related proteins (Cyclin-E1 and p-CDK2) levels were determined by Western blot analysis. The β-actin probe served as protein-loading control. The statistical analysis of the data was carried out by use of ANOVA test. ^*^P < 0.05, ^**^P < 0.01, ^***^P < 0.001 compared to N (untreated groups). N.S.; not significant (each experiment, n = 3). The error bars represent the standard error.

The WST-1 assay showed no cytotoxicity (N.S.; not significant) from the AC 2 peptide (0.1–1 mg/mL) in human dermal papilla cells after incubation for 24 h (Fig. 2A). In a previous study, DHT suppressed human dermal papilla cell proliferation and induced apoptotic cell death^22,23^. Cells treated with DHT (1 mg/mL) showed cytotoxicity (Fig. 2B). However, cotreatment with DHT (1 mg/mL) and AC 2 (1 mg/mL) did not induce significant cytotoxicity to human dermal papilla cells (Fig. 2C). These data showed that the AC 2 peptide rescues human dermal papilla cells from DHT-related stress. A cell cycle arrest assay using flow cytometry of the AC 2 peptide and DHT showed the G1/S phase ratio (Fig. 2D). The cells treated with DHT (1 mg/mL) showed that the G1 phase increased by 58.6% and the S phase decreased by 9.8% (G1 phase arrest). However, cotreatment with DHT (1 mg/mL) and AC 2 (1 mg/mL) induced cell cycle progression. The G1 phase increased by 38.9%, and the S phase decreased by 13.2%. In addition, cyclin-E1 and p-CDK2, which are crucial for the transition from the G1 phase to the S phase, are regulated by treatment with AC 2 (Fig. 2E).

### The AC2 peptide rescued human dermal papilla cells from DHT-induced stress by strengthening mTOR-raptor interactions and inhibiting autophagy and apoptosis

**Figure 3:**
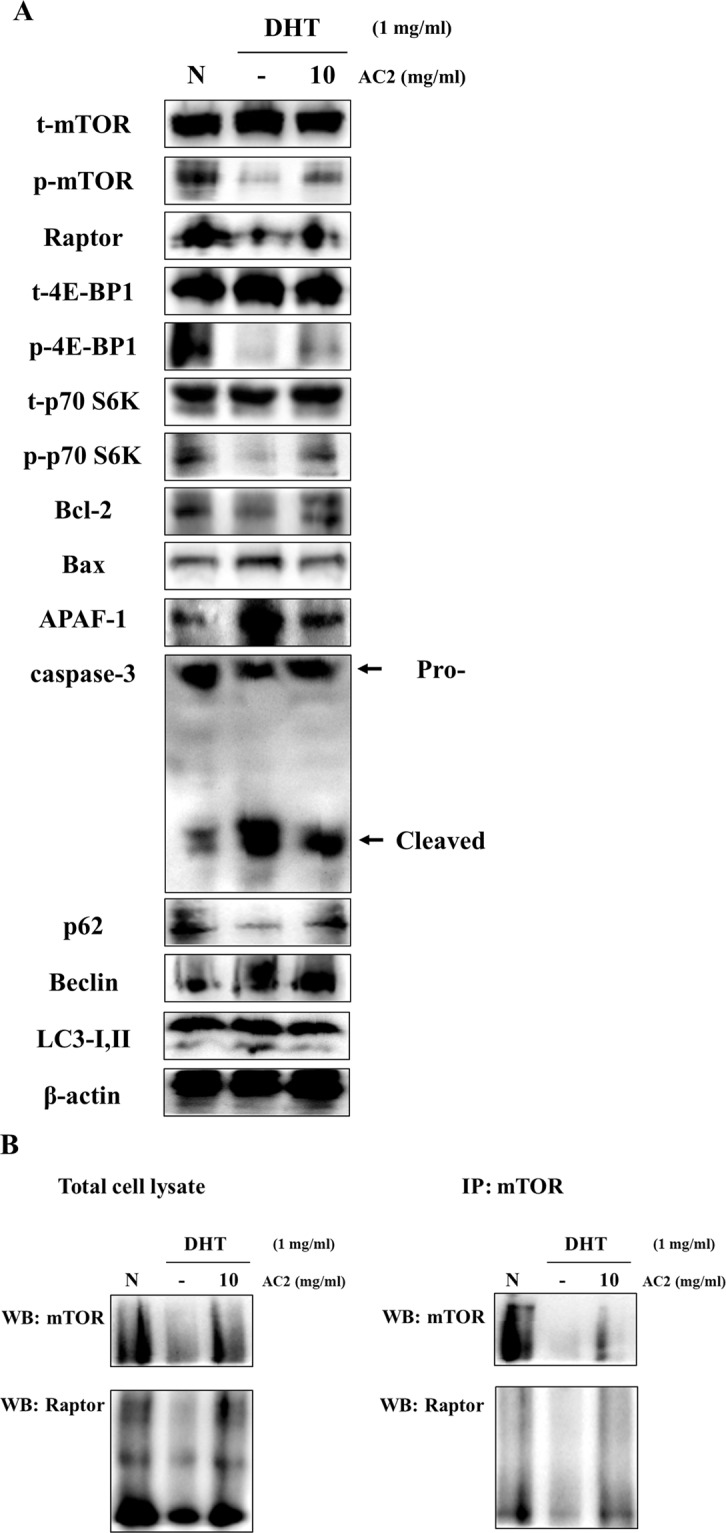
AC2 peptide rescue the Human dermal papilla cell from DHT-induced stress through strengthening mTOR-raptor interaction and inhibiting autophagy and apoptosis (**A**) Protein levels were determined by Western blot analysis. The β-actin probe served as protein-loading control. (**B**) Proteins interaction levels were investigated by immunoprecipitation (IP). Cell lysates were IP with antibody raised against mTOR. mTOR-raptor interactions were analyzed. N represents untreated cells.

**Figure 4:**
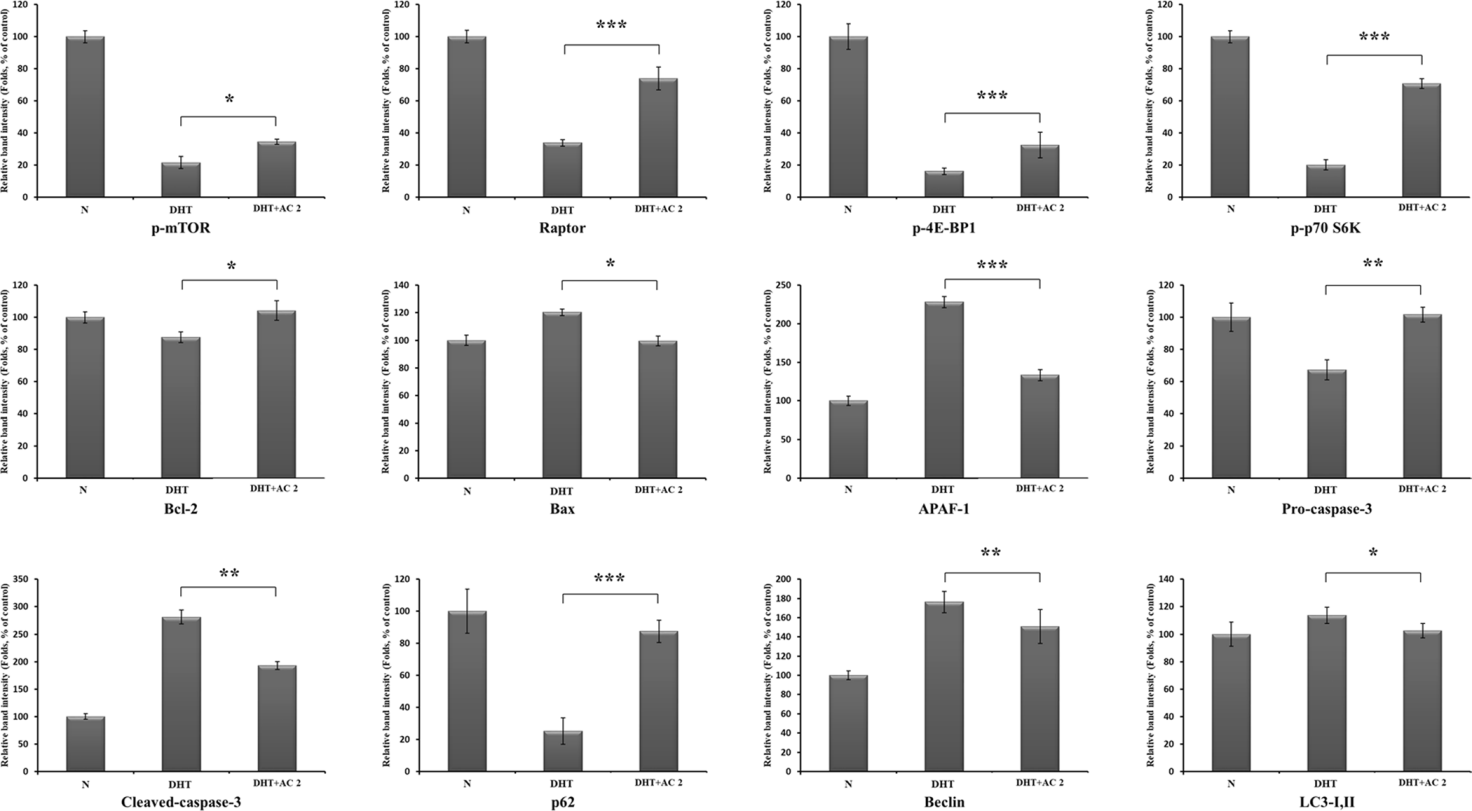
Relative band intensity of mTORC1 pathway-related protiens. The statistical analysis of the data was carried out by use of ANOVA test. ^*^P < 0.05, ^**^P < 0.01, ^***^P < 0.001 compared to N (untreated groups). N.S.; not significant (each experiment, n = 3) compared to DHT-treated group. ^#^*P* < 0.05, ^##^*P* < 0.01, ^###^*P* < 0.001 compared to DHT treated group. The error bars represent the standard error.

Figures 3 and 4 show the Western blot and immunoprecipitation assay results for AC 2 (10 mg/mL) and DHT (1 mg/mL) with the mTORC1 pathway for human dermal papilla cells. The analyses showed that DHT treatment significantly decreased the expression of phosphorylated-mTOR, 4E-BP1, p70 S6K and raptor in human dermal papilla cells. DHT treatment induces the cleavage of mTORC1 (mTOR-raptor interaction), initiating apoptosis and autophagy in the mitochondria and membranes of cells. However, DHT and AC 2 cotreatment regulated mTORC1-related protein expression compared to the cells treated with only DHT.

### Detection of apoptosis and autophagy activities using flow cytometry

**Figure 5:**
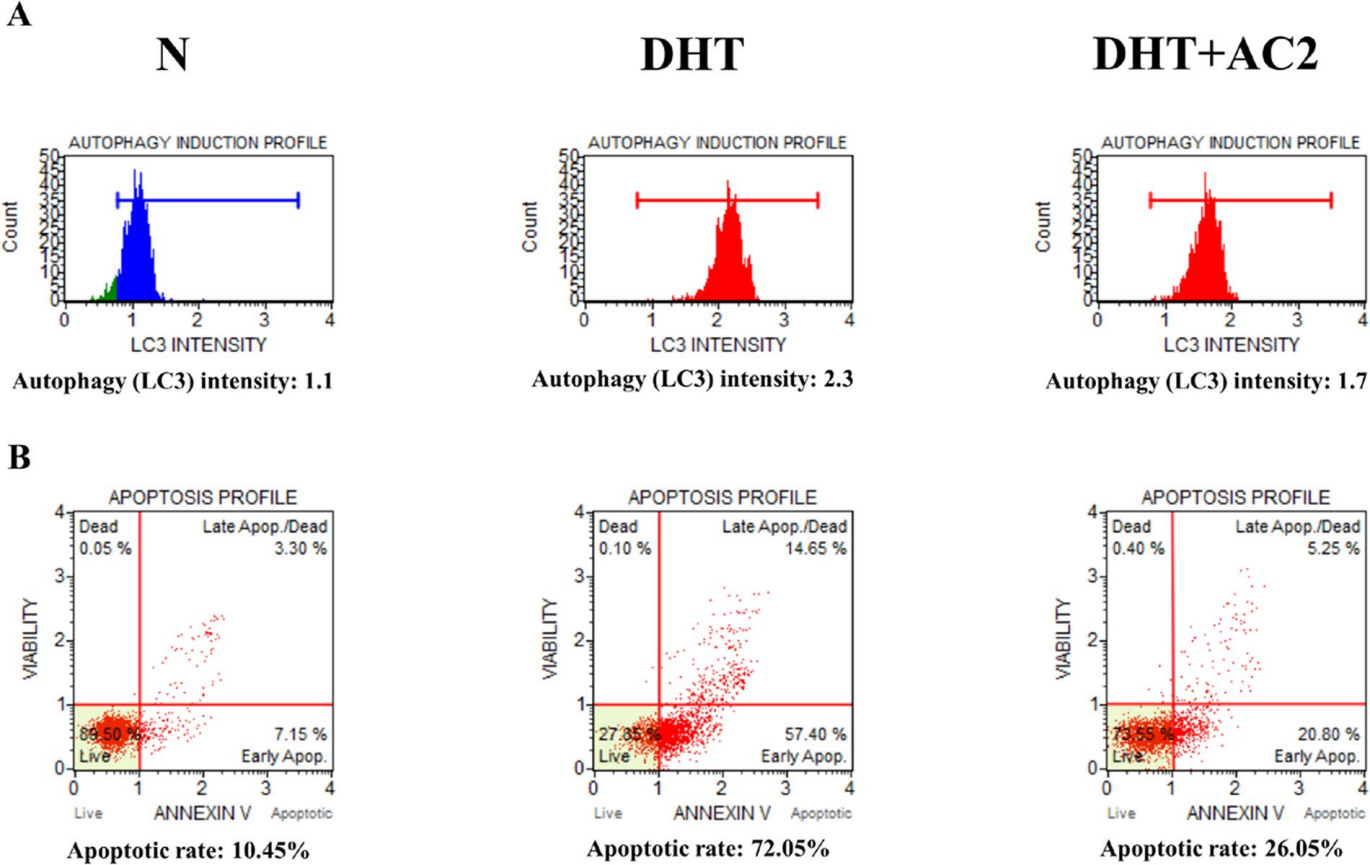
(**A**) Detection of Autophagy intensity in HDP using flow cytometry. (**B**) Detection of apoptotic rate in HDP using flow cytometry.

Flow cytometry is an effective technique used to detect the physical and chemical characteristics of a population of cells. Flow cytometric analysis found that when cells were treated with DHT, the number of autophagy- and apoptosis-induced cells were significantly increased compared to those without treatment. However, DHT and AC 2 cotreatment rescued the cells by inhibiting autophagy and apoptosis (Fig. 5a,b)

### Role of AC2 in the proliferation of *ex vivo* models

**Figure 6:**
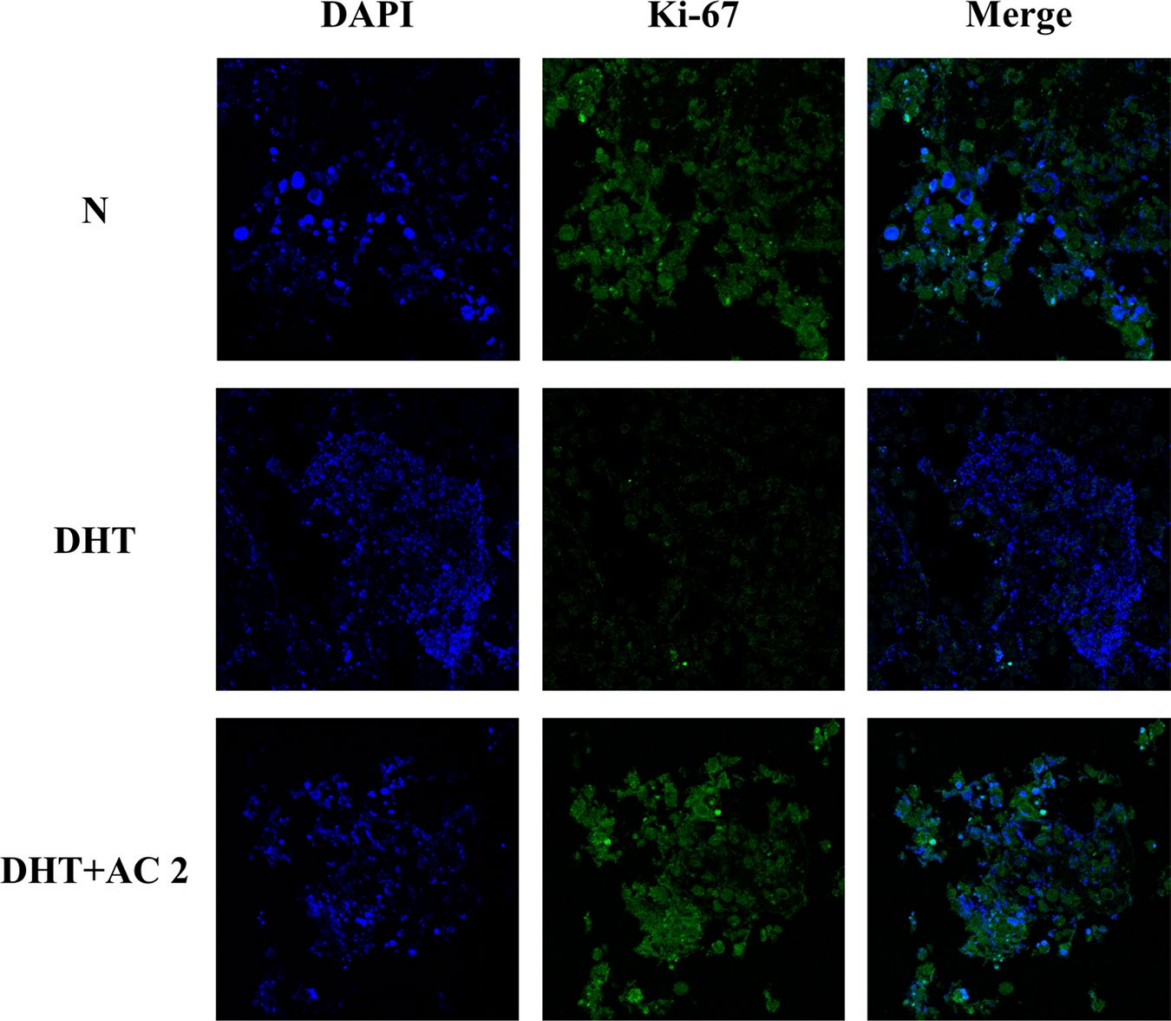
Fluorescence IHC *ex vivo*. The Organoytpic three-dimensional models using the Human dermal papilla cell were stained for DAPI, Ki-67 proteins. The sections were represented by a confocal microscope (Olympus, Japan).

In 3D culture studies, the morphology and types of expression involving major genes varied depending on the interaction between the cells and extracellular matrix. We investigated the effects of AC2 prior to clinical experimentation based on the differences in cellular drug responses in *ex vivo* models of human dermal papilla cells (Fig. 6). Treatment with only DHT inhibited Ki-67 expression (Ki-67 expression is strictly correlated with cell survival and proliferation and is widely used in routine pathological investigations) in the *ex vivo* model. However, Ki-67 expression in the AC2 and DHT cotreatment group increased compared with the DHT-treated group. These results showed that AC2 rescued the artificial tissue consisting of human dermal papilla cells from DHT-induced stress. The results indicate that AC2 can be used in human applications as a promising therapeutic candidate for human dermal papilla cells.

### 5. AC2 peptide synthetic mechanisms and investigation of their effect on human dermal papilla cells and the commercial potential as a novel medicine

**Figure 7:**
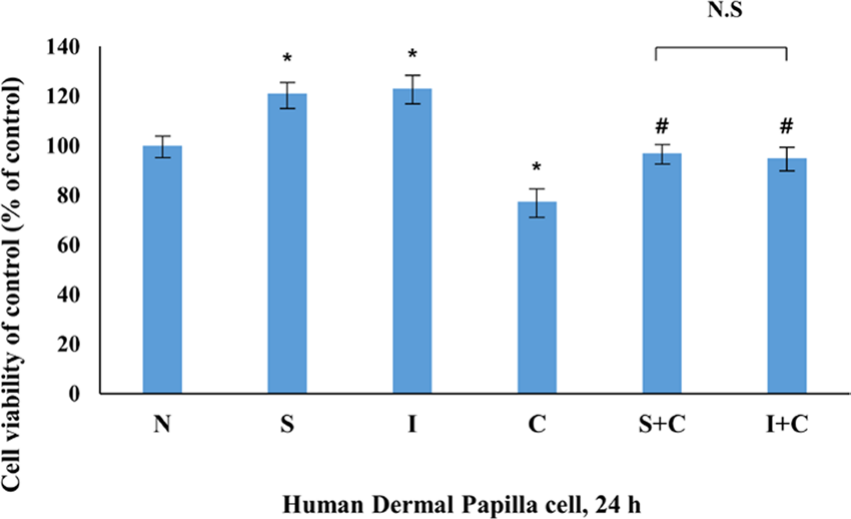
Cell viability was measured by WST-1 assay. Cells were treated to with synthesized and isolated AC2 peptide (10 mg/ml) and DHT (1 mg/ml) for 24 h. N; Negative control, S; Synthesized AC 2 peptide, I; Isolated from *Bacillus* treated *Trapa japonica Fruit*. C; DHT treated group. The statistical analysis of the data was carried out by use of ANOVA test. ^*^P < 0.05, ^**^P < 0.01, ^***^P < 0.001 compared to N (untreated groups). N.S.; not significant (each experiment, n = 3) compared to N (untreated groups). ^#^*P* < 0.05, ^##^*P* < 0.01, ^###^*P* < 0.001 compared to DHT treated group. The error bars represent the standard error.

As shown in the Methods section, the details of the AC 2 peptide synthetic mechanisms are as mentioned. The synthesized AC2 peptide showed similar effects to the isolated AC2 peptide from *Trapa japonica* fruit through various experiments (Fig. 7). Even with DHT treatment, the high concentration of the synthesized and isolated AC2 peptide (10 mg/ml) enhanced the proliferation of human dermal papilla cells. These results suggest that the synthesized AC2 peptide is an alternative to the expensive and difficult isolation and purification procedures.

## Discussion

Taken together, the data in this research suggest that treatment with the AC 2 peptide rescues DHT-treated human dermal papilla cells by inhibiting cleavage of the mTORC1 complex and suppressing autophagy and apoptosis. To the best of our knowledge, this research is the first attempt to improve the peptide contents of natural extracts by fermentation using two kinds of bacteria (*Bacillus methylotrophicus and Bacillus subtilis*)^24,25^.

Moreover, the research was conducted with various experimental techniques to improve the purification of the peptide, such as liquid chromatography. As the fermentation and isolation process progresses, we confirmed that the cytotoxicity decreased and the peptide content increased. Additionally, we found and isolated the AC 2 peptide from many *Bacillus*-treated *Trapa japonica* fruit peptides. These results showed the importance of the fermentation and isolation processes using liquid chromatography. Then, we analyzed the structure of the AC 2 peptide and demonstrated whether it can be synthesized as a drug. In addition, we confirmed the bioactive mechanism of the AC2 peptide on DHT-treated stressed human dermal papilla cells through various experiments.

mTOR is a serine/threonine kinase belonging to the PI3K-related kinase group and comprises two complexes: mTORC1 and mTOR complex 2 (mTORC2)^26^. The three core components of mTORC1 include mTOR, regulatory protein associated with mTOR (raptor), and mammalian lethal with Sec. 13 protein 8, which is also known as GßL. Raptor promotes binding between the mTOR signaling motif structure and mTORC1, which are commonly found in mTORC1 substrates^27^. Raptor acts as a scaffold protein inside mTORC1, maintaining the state of the complex. It initiates the translation of 4E-binding protein 1 (4E-BP1) bound to p70 S6 kinase 1 (p70 S6K1), activating its phosphorylation by mTOR and inducing mTORC1-mediated protein synthesis^28^.

Raptor binding is required for normal positioning of mTORC1 in the organelle. mTORC1 is essential for cell proliferation and viability, and when the complex is disrupted by DHT and stress, this may affect the proliferation of dermal papilla cells via apoptosis, cell cycle arrest, and autophagy^29^. In dermal papilla cells, autophagy occurs at a basal level for the continued metabolism of intracellular components^30,31^. However, mTORC1 inhibits autophagy and apoptosis while maintaining the cell cycle. The cleavage of mTORC1 and raptor by DHT affects cell viability and activates autophagy to generate energy for cell viability and the reuse of nutrients^32^. Moreover, dephosphorylation of mTOR affects its downstream targets and thereby facilitates apoptosis and autophagy initiation. Dephosphorylation and stress induced by DHT accelerate apoptosis and autophagy. During this process, beclin 1, light chain (LC) 3- I, and LC II are generated to form an autophagosomal membrane. Simultaneously, p62 (sequestosome 1) binds to a ubiquitin-associated domain to translocate defective proteins or cell organelles to the autophagosomal membrane. In addition, excessive autophagy induces apoptosis and not protection. Apoptosis is activated by cleavage of mTORC1 or excessive autophagy and rarely induces immunity, unlike necrosis^33^. When the equilibrium of the Bcl–Bax complex is disturbed on the outer membrane of mitochondria due to stress, pore formation occurs in the outer membrane, triggering leakage of cytochrome C into the cytoplasm. The leaked cytochrome C binds to apoptosis protease-activating factor-1, resulting in the synthesis of apoptotic protease activating factor-1 (APAF-1). Cleaved caspase-3 (activated by APAF-1) activates the caspase cascade in the cytoplasm, which further induces apoptosis. In our study, we found that the cleavage of the mTORC1 complex is activated during DHT treatment. The AC 2 peptide, which acts by specifically inhibiting the cleavage of the mTORC1 complex, is recognized as a biomedicine that inhibits DHT-related stress in human dermal papilla cells.

We have demonstrated that stress in dermal papilla cells induced by DHT can be reduced by the AC2 peptide, suggesting strong pharmacological effects of the AC2 peptide. Furthermore, we verified the effects of the AC2 peptide using a three-dimensional cell culture model. Studies have reported large differences in drug responsivity in three-dimensional cell culture models since they not only incubate the cells in three dimensions but also create conditions similar to the physiological environment^34^. In particular, a three-dimensional culture model revealed that the morphology and types of expression involving the major genes and proteins vary depending on the interaction between the cells and extracellular matrix^35^. We investigated the effects of AC2 again prior to clinical experimentation using the cellular drug responses in 3D cell culture models. Furthermore, we also synthesized the AC2 peptide as an alternative to the expensive and difficult isolation and purification procedures. We confirmed the potential of the AC2 peptide as a biomedicine and verified the similar effects of the synthetic AC2 peptide to those of the isolated and purified AC2 peptide.

The emerging role of the AC 2 peptide is an important issue and plays a key role in a new cell therapeutic approach.

However, the exact function of the AC 2 peptide remains controversial, and its precise mechanism related to preclinical and clinical trials remains to be elucidated. In conclusion, this study systematically investigated the data for the AC 2 peptide from *Bacillus*-treated *Trapa japonica* fruits, aiming to provide clarity surrounding the role of cell therapy. Our results demonstrate that the AC 2 peptide from *Bacillus*-treated *Trapa japonica* fruits rescues DHT-treated human dermal papilla cells and its effects are mediated through the mTORC1 signaling pathway.

## Materials and Methods

### Reagent

WST-1 assay kit was purchased from Daeillab (Daeillab, Korea). and dihydrotestosterone (DHT) were purchased from Sigma Aldrich (Sigma Aldrich, USA). Specific antibodies such as Bcl-2, Bax, APAF-1 was purchased from Santa Cruz Biotechnology (Santa Cruz Biotechnology, USA). Specific antibodies such as t-mTOR, p-mTOR, LC3, p62 Cyclin E1, p-cdk2, t-p70 S6K, p-p70 S6K1, t-4E-BP1, p-4E-BP1 were obtained from Cell Signaling Technology (Beverly, USA). and Specific antibodies such as β-actin and Active-caspase-3 antibodies were purchased from Abcam (Cambridge, USA). Muse Cell Cycle Kit (MCH100106) and Muse Cell Analyzer (PB4455ENEU) were purchased from Millipore (EMD Millipore Corporation, Germany).

### Extraction and fermentation of trapa japonica fruit

*Trapa japonica* fruit (Taxa and representative voucher specimen number: KP255650) was grown in China and purchased from BS corporation (BS corporation, KOREA). The hot water extract of *Trapa japonica* fruit was heated at 37 °C with distilled water, followed by concentration and vacuum filtration in the water bath at 37 °C. The hot water extract of *Trapa japonica* fruit (10,000 ppm) was fermented using *Bacillus methylotrophicus* from MRS agar medium and *Bacillus subtilis* from MRS agar medium (respectively 1.0 × 10^6^ cfu/ml). *Trapa japonica* fruit and medium were mixed with glucose, yeast extract and soytone. The final fermentation of extract was performed by mixing microorganisms (*Bacillus methylotrophicus* and *Bacillus subtilis)*. It was fermented in a 37 °C fermenter for 72 h, followed by filtration with a 0.2 μm filter and centrifugation to remove the microorganisms completely. The fermented *Trapa japonica* fruit extract was sequentially separated into hexane, CH2Cl2, EtOAc, N-BuOH fractions and water layers. Subsequently, the water layer was separated using the silica gel column (Waters, USA) to obtain the six fractions. The six fractions were resolved using a Sephadex-LH20 column (Waters, USA). Fractions 1–6 were obtained via HPLC prep HPLC Xterra C18 ODS column (Waters, USA). The fraction was refined using HPLC (Waters, USA). The mobile phase consisted of water:ACN:MeOH solution (water:MeOH = 90:10, 80:20, 70:30, and 0:100) pumped at a flow rate of 1 mL/min. The fraction 2 was chromatographed over a RP silica gel column (Waters, USA), and eluted using the water-MeOH gradient and monitored via TLC (thin layer chromatography) to separate the five fractions. Later, single compounds 1 to 6 were isolated from the fraction 2–2 using preparative HPLC. The compound 1 was concentrated under vacuum, and freeze-dried completely. The compound 1 powder was designated as ‘AC2 peptide’ and dissolved in distilled water.

### Fractionation and isolation of AC2 peptide from Bacillus/Trapa japonica fruit and NMR profiling

Ferment *Trapa japonica fruit* was further fractionated with n-hexane, CH_2_Cl_2_, EtOAC, n-BuOH and then with water. Five fractions were obtained, respectively. After confirming the protein content, the effective Water fraction was concentrated under reduced pressure using a rotary vacuum concentrator (Dlab, RE100-pro). And then subjected to column chromatography on columns of silica gel (70~230 mesh, Merck art 4 * 20 cm) using a Water–MeOH gradient (Water: MeOH = 90:10, 80:20, 70:30, 0:100) and Sehpadex LH20 column chromatography (MeOH) to afford five fractions (Fr. 1~5). Fraction 2 was chromatographed over a RP silica gel column, eluting with a Water-MeOH gradient and monitored by thin layer chromatography (TLC Merck Art.) to separate five fractions (Fr. 2–1~2–5). After that, single compounds 1 to 6 were isolated from fraction 2-2 by high-performance liquid chromatography (HPLC) preparation. HPLC analysis was performed on a Waters 2690 Separation module and a Waters 2487 Dual Absorbance Detector. The analytical column **(**Xterra C18 ODS, 0.5 × 250 nm) was packed with Lichrospher 100RP-18 (15μm, Merck Co,) Compound 1 White amorphous powder; EI-MS m/z 303.13 [M] + 1H-NMR (D2O, 600 MHz) ▯1.859 (4 H, m, 13, 6-CH2), 1.941 (3 H, s, 15-CH3), 2.025 (1 H, m, 7-CH2), 2.138 (1 H, m, 7-CH2), 2.439 (2 H, m, 14 S-CH2), 3.818 (2 H, s, 2-CH2) 4.321,4.394 (1 H, m, 8-CH), 13C-NMR (D2O, 150 MHz) using Bruker Avance II^+^-500 FT-NMR Spectrometer (Bruker, USA).

### Cell culture

Human dermal papilla cells were obtained from CEFO (CEFO, KOREA). Human dermal papilla cells were grown in DMEM medium (Hyclone, USA) containing 1% antibiotics (100 U/ml penicillin and 100 mg/streptomycin) and 10% Fetal bovine serum (Hyclone, USA) and at 37 °C in a 5% CO_2_ atmosphere. Cells were suspended by Trypsin-EDTA (Hyclone, USA) every 2 days.

### WST-1 assay

Cells were seeded at 3.8** × **10^5^ cells/ml in a 12-well plate for 24 hours and were incubated with AC2 peptide (0.1–10 mg/ml) and DHT (dihydrotestosterone, 1 mg/ml) for various hours. Following incubation with the AC 2 peptide and DHT, the cells were incubated with a 100 μl/ml Wst-1 solution (Daeillab, Korea) for 60 min. Then, the optical densities of the solutions were quantified at a 450 nm wavelength by using a FLUOstar Omega (BMG labtech, Germany).

### Determination of cell cycle

Cells were seeded at 9.5** × **10^5^ cells/ml in a 6-well plate. After 24 hours incubation, cells were treated with AC2 peptide (0.1–10 mg/ml) and DHT (dihydrotestosterone, 1 mg/ml) for 24 hours. Following incubation, the cells were resuspended with PBS. And slowly add 200 μL of pre-cold 70% ethanol. After incubate for 24 hours at −20 °C, the fixed cells were mixed with 200 μL of premixed reagent including the nuclear DNA intercalating stain PI (propidium iodide) and RNAse and incubated for 30 minutes at room temperature in the dark. Then, the stained cells were analyzed in Muse Cell Analyzer (Merck Millipore Co.**)**. the stained cells at different stages of the cell cycle, based on differential DNA content.

### Western blotting

Cells were seeded at 1** × **10^6^ cells/ml in a 6-well plate. After incubation, cells were treated with AC2 peptide (0.1–10 mg/ml) and DHT (dihydrotestosterone, 1 mg/ml) for 24 hours. After a 24 hours, cells were rinsed with pre-cold PBS, scraped with a RIPA buffer (Sigma Aldrich, USA) with Halt Protease and Phosphatase inhibitor cocktail (ThermoFisher, USA) and sonicated using Ultra sonicator (KP Tech., Korea) subjected to the western blot analysis. Protein quantification was performed using a Bradford assay and 50 μg of protein were loaded per lane. Primary antibodies (t-mTOR (1: 2,000; cat. No. #2972), p-mTOR (1: 2,000; cat. No. #2971), Raptor (1: 2,000; cat. No. 24C12), t-4E-BP1 (1: 2,000; cat. No. #9452), p-4E-BP1 (1: 2,000; cat. No. 236B4), t-p70-S6K (1: 2,000; cat. No. #9202), p-p70-S6K (1: 2,000; cat. No. #9234 T), Bcl-2 (1: 2,000; cat. no. #4223), Bax (1: 2,000; cat. no. #2772), APAF (1: 2,000; cat. No. #G1310), caspase-3 (1: 1,000; cat. no. ab4051), p62 (1: 2,000; cat. No. #88588), caspase (1: 2,000; cat. No. #G1310), Beclin-1 (1: 2,000; cat. no. #3738), LC-3 (1: 1,000; cat. #3868), Cyclin E1 (1: 2,000; cat No. 20808), p-Cdk2 (1: 2,000; cat ab76146) and β-actin (1: 2,000; cat. no. 3700)) reacted overnight at 4 °C and secondary antibodies (Anti-mouse IgG, HRP-linked Antibody #7076 and Anti-Rabbit IgG, HRP-linked Antibody #7074) reacted for 120 min at 4 °C. Western blotting was repeated at least times for each experiment.

### Immunoprecipitation

Cells were seeded at 1** × **10^6^ cells/ml in a 6-well plate. After a 24 hours incubation, cells were treated with AC2 peptide (0.1–10 mg/ml) and DHT (dihydrotestosterone, 1 mg/ml) for 24 hours. After a 24 hours, cells were rinsed twice with ice cold PBS, scraped with a RIPA buffer (Sigma Aldrich, USA) with Halt Protease and Phosphatase inhibitor cocktail (ThermoFisher, USA). Cells were centrifuged at 14,000 x g for 5 minutes to obtain cell lysates. Cell lysates were incubated with anti-mTOR antibody (Cell signaling, USA) and reacted overnight at 4 °C. Then, pretein A/G PLUS-agarose (Santa Cruz Biotechnology, USA) was added to the cell lysate and reacted overnight at 4 °C. The next day, the beads were washed with ice cold PBS three times and heated with SDS sample buffer for 5 minutes at 100 °C. The samples were subjected to the western blot analysis. Western blotting was repeated at least times for each experiment.

### Organotypic 3D cell culture model

The orgranotypic 3D culture (OTC) is followed Hiro Nakagawa, MD, PhD protocol^36^. the methods are followed.

Matrix (consist of Fetal bovine serum (Hyclone, USA) and DMEM medium (Hyclone, USA), type I collagen) is prepared along with Human Dermal papilla cells on overnight. Human Dermal papilla cells were seeded on 4 days, grown in a EP2 medium (consist of DMEM, F12, Progesterone, L-Glutamine, Hydrocortisone, Newborn Calf Serum etc.), and then exposed for 5 days to reduced volume of medium. Then, change the medium to EP3 medium (EP2 medium without Progesterone) for 5 days. Incubated for 10 days with AC2 peptide (0.1–10 mg/ml) and DHT (dihydrotestosterone, 1 mg/ml) at 37 °C in a 5% CO_2_ atmosphere.

### Immunohistochemistry (IHC)

The organotypic 3D cell culture models were fixed in 10% Neutral buffered formalin for overnight and incubated with PBS at 4 °C for 2 days. Then, the models were sectioned and embedded in paraffin. The embedded sections were fixed in cold-acetone for 10 min. the sections were treated with 3% H_2_O_2_ and blocked with 10% normal goat serum (Invitrogen, USA). Then, the sections were incubated with a Ki-67 primary antibody (1:400; cat No. #9027) overnight at 4 °C. On the second day, the sections were incubated with an Alexa Flour 488-conjugated secondary antibody (1:1000; cat No. #4412) and mounted using Antifade mounting medium with DAPI (VECTASHIELD, UK) for 2 hours. the sections were observed using a confocal microscope (Olympus, Japan).

### AC2 peptide synthetic mechanisms

AC2 synthesized three amino acids (Fmoc Serine, Glycine and Proline) through the following process. First, three amino acids were inflated and cleaned using 2-Chlorotrityl chloride resin and methylene chloride (MC). Then, after reacting with DMF (Dimethylamide) and DIPEA (Dimethylamine) in amino acids, the primary amino acid was attached by reacting with a De-Blocking solution. next, dissolving with HOBt containing DMF and mixing with HBTU added DIPEA to attach another amino acid. The synthesized resin was concentrated with TFA (Tripluoroacetic acid) and MC. The synthesis process was repeated to react with TFA and distilled water in concentrated resins, distributed in ether, and dried. Dried powder was dissolved in distilled water to synthesize AC2 peptide with a purity of 95% or higher using prep LC.

### Statistical analysis

All the experiments were repeated at least three times and analyzed using t-tests (SPSS 20.0, USA). p <0.05 was considered to indicate a statistically significant difference.

### equipment and settings

All figure images acquisition tools and image processing using PowerPoint (Microsoft, USA).

Images gathered at different times or from different locations not be combined into a single image.

Image processing (such as changing brightness and contrast) is appropriately used only when it is applied equally across the entire image and is applied equally to controls.

All figure images complied with the integrity policies

## Supplementary Information

Supplementary file

## Data Availability

All data generated or analyzed during this study is included in this article.
