# Author Correction: The peptide AC 2 isolated from *Bacillus*-treated *Trapa japonica* fruit extract rescues DHT (dihydrotestosterone)-treated human dermal papilla cells and mediates mTORC1 signaling for autophagy and apoptosis suppression

**DOI:** 10.1038/s41598-020-65542-8

**Published:** 2020-05-19

**Authors:** Gun He Nam, Kyung-Jo Jo, Ye-Seul Park, Hye Won Kawk, Je-Geun Yoo, Jin Dong Jang, Sang Moon Kang, Sang-Yong Kim, Young-Min Kim

**Affiliations:** 1 Department of Biological science and Biotechnology, College of Life science and Nano technology, Hannam University, 1646 Yuseong-daero, Yuseong-gu, Daejeon 34054 South Korea; 2 Doori Cosmetics Co.,Ltd., 11F Galaxy Tower, 175, Saimdang-ro, Seocho-gu, Seoul South Korea; 3 ANPEP INC. 13, Oksansandan 1-ro, Oksan-myeon, Heungdeok-gu, Cheongju-si, Chungcheongbuk-do Republic of Korea; 4 Department of Food Science & Bio Technology, Shinansan University, Daehakro Danwon-gu, Ansan-city, Gyenggi-do South Korea

Correction to: *Scientific Reports* 10.1038/s41598-019-53347-3, published online 15 November 2019

This Article contains errors.

As a result of a data mix-up during preparation of the figures, data for Beclin from Figure 3E is also presented as data for p-CDK2 from Figure 2E. The correct Figure 2E is shown below as Figure 1.

**Figure 1:**
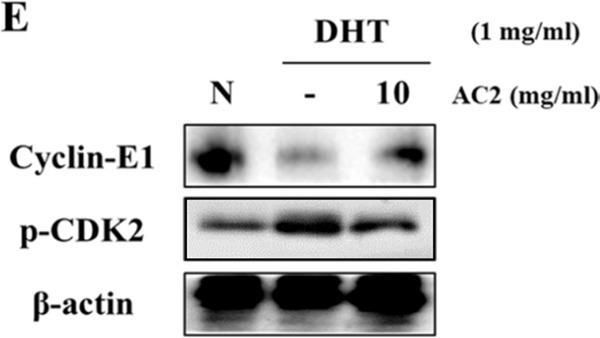
.

The correct original membrane for p-CDK2 is shown below as Figure 2.

**Figure 2:**
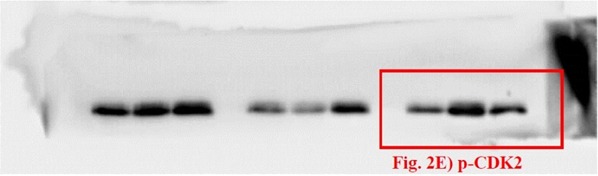
.

This correction does not affect the conclusions of the Article.

