# Supplementary material for: The peptide AC 2 isolated from Bacillus-treated Trapa japonica fruit extract rescues DHT (dihydrotestosterone)-treated human dermal papilla cells and mediates mTORC1 signaling for autophagy and apoptosis suppression
Source: Sci Rep. 2019 Nov 15;9:16903. doi: 10.1038/s41598-019-53347-3 (PMC6858360; doi:10.1038/s41598-019-53347-3)
Supplement: Supplementary file 1 — Supplementary file [file 41598_2019_53347_MOESM1_ESM.pdf]

Figure 3 supplementary data

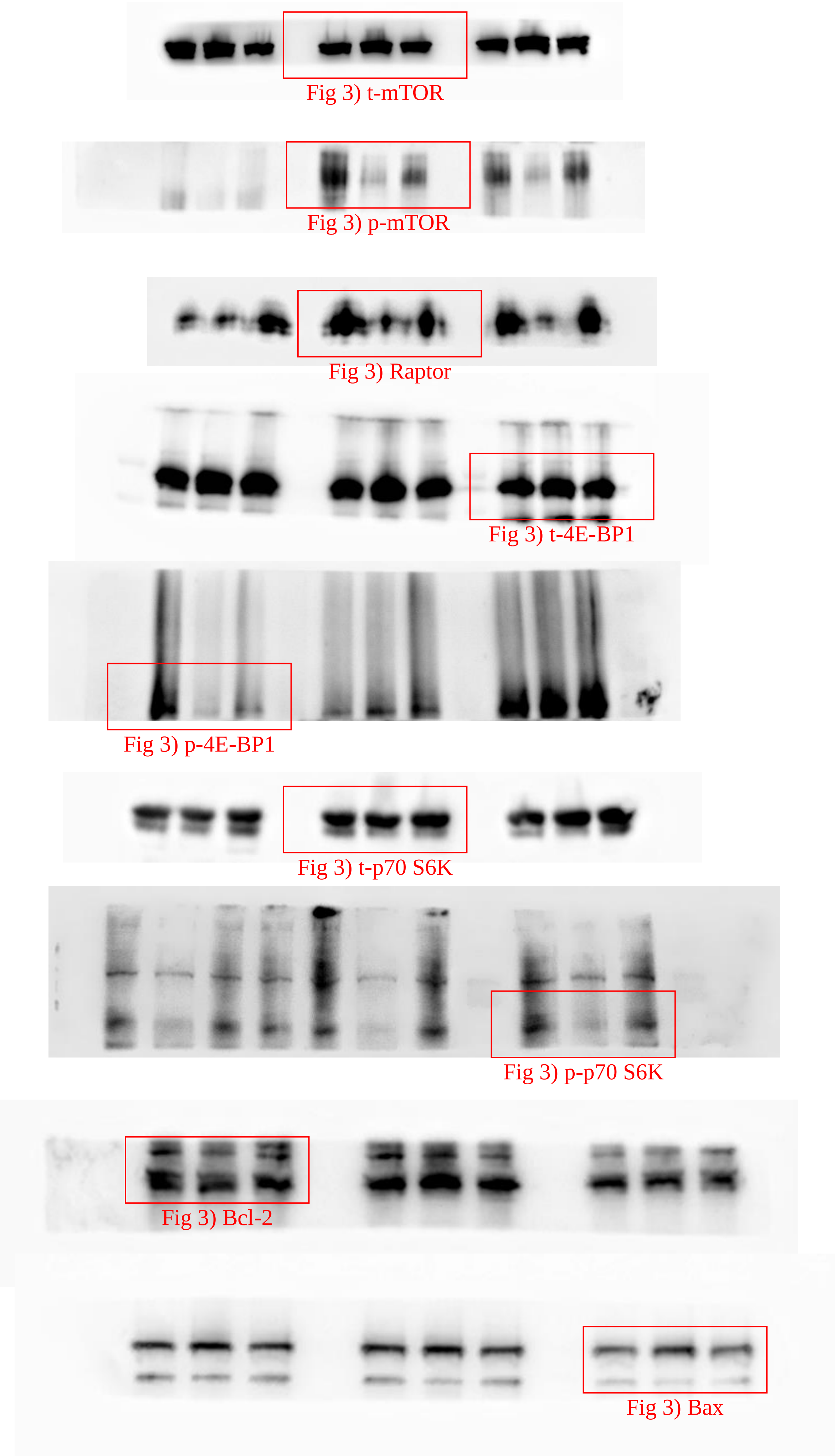

Figure 3 supplementary data

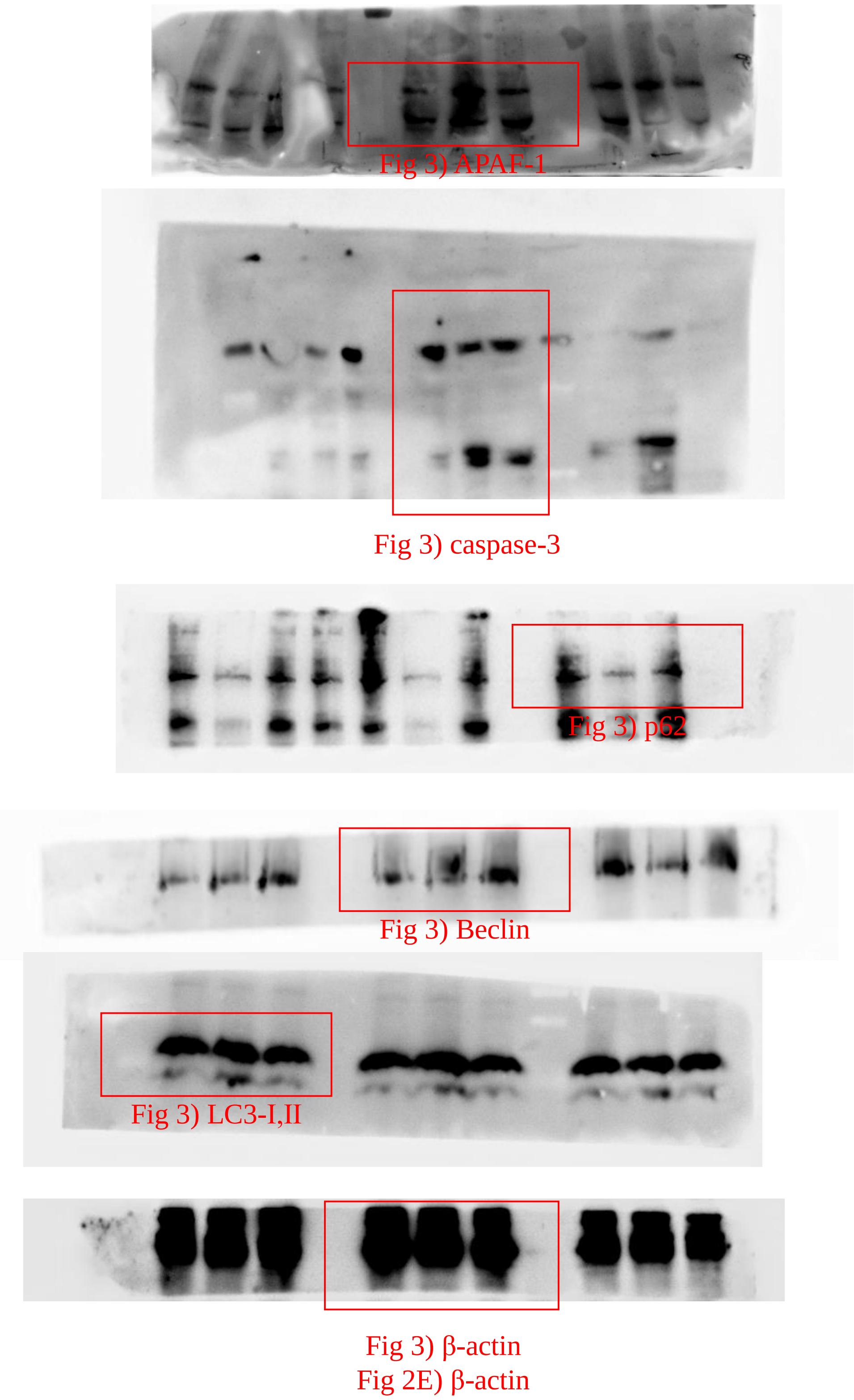

Figure 2E supplementary data

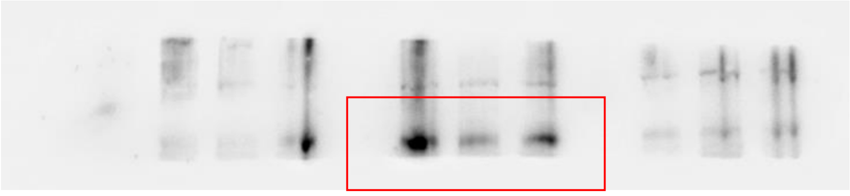

Fig 2E) Cyclin-E1

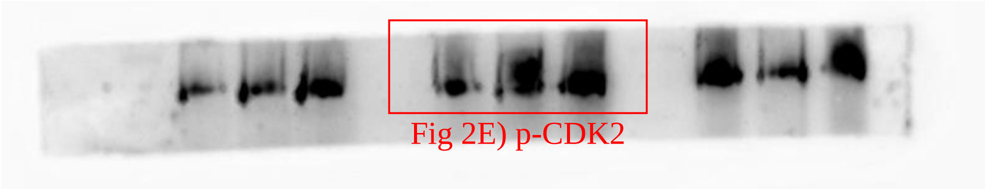

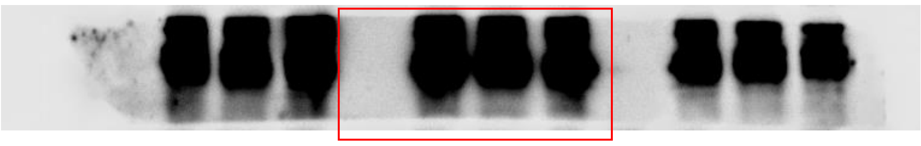

Fig 3) β-actin
Fig 2E) β-actin

Figure 3 supplementary data

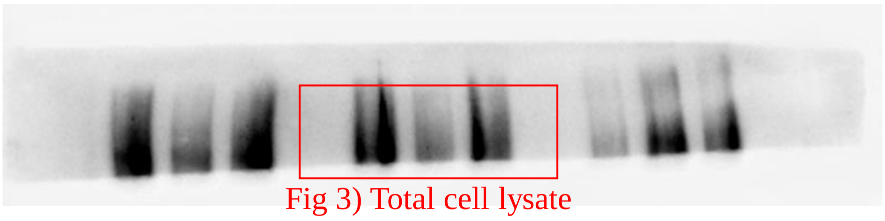

Fig 3) Total cell lysate
WB:mTOR

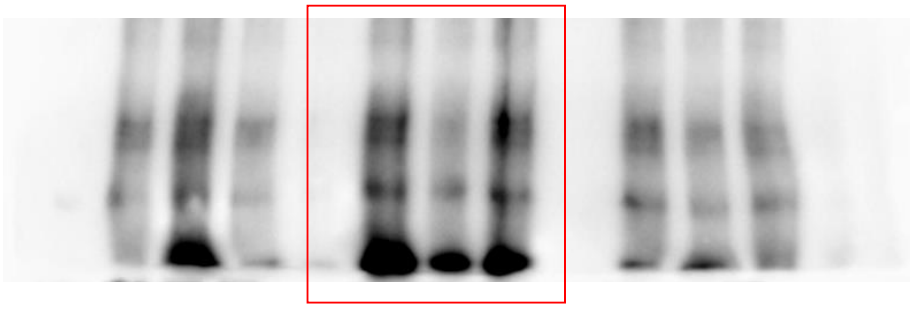

Fig 3) Total cell lysate
WB:Raptor

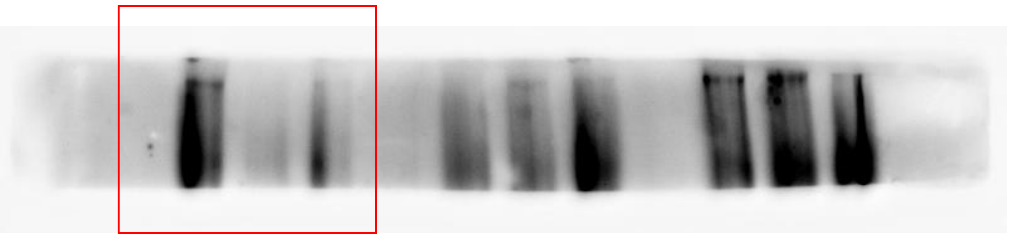

Fig 3) IP:mTOR
WB:mTOR

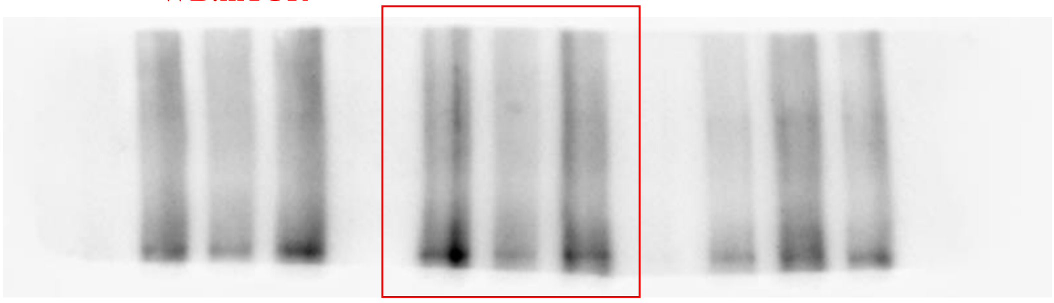

Fig 3) IP:mTOR
WB:Raptor
